# Mechanical and Thermal Properties of Montmorillonite-Reinforced Polypropylene/Rice Husk Hybrid Nanocomposites

**DOI:** 10.3390/polym11101557

**Published:** 2019-09-25

**Authors:** Khaliq Majeed, Ashfaq Ahmed, Muhammad Saifullah Abu Bakar, Teuku Meurah Indra Mahlia, Naheed Saba, Azman Hassan, Mohammad Jawaid, Murid Hussain, Javed Iqbal, Zulfiqar Ali

**Affiliations:** 1 Department of Polymer Engineering, Faculty of Chemical and Energy Engineering, Universiti Teknologi Malaysia, 81310 Johor, Malaysia; azmanh@cheme.utm.my; 2 Department of Chemical Engineering, COMSATS University Islamabad, Lahore Campus, Islamabad 45550, Pakistan; ashfaqengr97@gmail.com (A.A.); drmhussain@cuilahore.edu.pk (M.H.); zulfiqar.ali@cuilahore.edu.pk (Z.A.); 3 Faculty of Integrated Technologies, Universiti Brunei Darussalam, Jalan Tungku Link, Gadong BE 1410, Brunei; saifullah.bakar@ubd.edu.bn; 4 School of Information, Systems and Modelling, Faculty of Engineering and Information Technology, University of Technology Sydney, Ultimo NSW 2007, Australia; TMIndra.Mahlia@uts.edu.au; 5 Laboratory of Biocomposite Technology, Institute of Tropical Forestry and Forest Products (INTROP), Universiti Putra Malaysia, Serdang, 43400 Seri Kembangan, Selangor, Malaysia; Naheedchem@gmail.com (N.S.); jawaid@upm.edu.my (M.J.); 6 Department of Chemical Engineering, Khwaja Fareed University of Engineering and Information Technology, Abu Dhabi Road, Rahim Yar Khan 64200, Pakistan; javed.iqbal@kfueit.edu.pk

**Keywords:** nanoparticles, polypropylene, composites, mechanical properties, thermal properties

## Abstract

In recent years, there has been considerable interest in the use of natural fibers as potential reinforcing fillers in polymer composites despite their hydrophilicity, which limits their widespread commercial application. The present study explored the fabrication of nanocomposites by melt mixing, using an internal mixer followed by a compression molding technique, and incorporating rice husk (RH) as a renewable natural filler, montmorillonite (MMT) nanoclay as water-resistant reinforcing nanoparticles, and polypropylene-grafted maleic anhydride (PP-g-MAH) as a compatibilizing agent. To correlate the effect of MMT delamination and MMT/RH dispersion in the composites, the mechanical and thermal properties of the composites were studied. XRD analysis revealed delamination of MMT platelets due to an increase in their interlayer spacing, and SEM micrographs indicated improved dispersion of the filler(s) from the use of compatibilizers. The mechanical properties were improved by the incorporation of MMT into the PP/RH system and the reinforcing effect was remarkable as a result of the use of compatibilizing agent. Prolonged water exposure of the prepared samples decreased their tensile and flexural properties. Interestingly, the maximum decrease was observed for PP/RH composites and the minimum was for MMT-reinforced and PP-g-MAH-compatibilized PP/RH composites. DSC results revealed an increase in crystallinity with the addition of filler(s), while the melting and crystallization temperatures remained unaltered. TGA revealed that MMT addition and its delamination in the composite systems improved the thermal stability of the developed nanocomposites. Overall, we conclude that MMT nanoclay is an effective water-resistant reinforcing nanoparticle that enhances the durability, mechanical properties, and thermal stability of composites.

## 1. Introduction

The ever-growing demand for food, energy, and building services; increasing comfort levels; and strong dependence on fossil fuels and their derivatives are badly affecting our ecosystem. Ozone layer depletion, climate change, and the deleterious effects of fossil-fuel-based plastics on marine life and their inherent nonbiodegradability are examples of the far-reaching consequences that fossil fuels have on our ecosystem [1,2,3,4]. These issues have motivated researchers to explore renewable energy and environmentally friendly materials. Studies on the potential applications of renewable and sustainable resources to replace fossil derivatives have achieved various levels of success [5,6,7,8,9,10,11]. The growing interest in green materials due to mounting ecological and environmental issues has also triggered the investigation of natural fibers as potential reinforcing fillers for polymeric materials. Natural fibers possess remarkable mechanical properties and are renewable, ecofriendly, and greatly susceptible to microorganisms [12,13]. Thermoplastic polymers and their fiber-reinforced composites are of great interest, as they have a diverse range of indoor and outdoor applications [14,15,16] and can be used as load bearing, structural, and decorative materials. A wide range of thermoplastic composite materials with natural fibers as reinforcing agents has been reported, and their applications in the automobile, construction, and other industries have shown encouraging results [17,18].

Among various industrial by-products of natural fibers, rice husk (RH) is an abundantly available resource. It is the nonedible natural sheath around a rice grain (paddy) that forms during its growth and provides protection for the paddy against termites and microbial attacks [19,20,21]. About 0.23 t of RH is removed and separated during the processing of every ton of kernels [21,22]. Due to annual renewability of rice, large quantities of its by-product (RH) are also produced every year. However, this by-product does not have any commercial use. Silica, a major constituent of RH, has already been suggested as a reinforcing filler for various polymeric matrices [23,24] to enhance mechanical properties. Another study comparing RH composites with wood-fiber composites revealed that RH composites have better resistance to termites and other organisms [25]. The possible advantages of incorporating renewable RH as a reinforcing filler for use in composite and green nanocomposite development has created a new platform in the polymer industries.

However, the potential benefits of incorporating natural fiber reinforcements in composites depend mainly on their structural and mechanical properties, which are influenced by their application environment. Despite the many advantages of using natural fibers as reinforcements and fillers, their dimensional instability, due to their hydrophilic character and susceptibility to rotting, restrict their use in outdoor and structural applications. The poor water resistance and dimensional instability of natural fibers relate to their major constituent, cellulose. The excessive availability of free hydroxyl groups in cellulose is a major cause of its swelling, as these groups have the potential for hydrogen bonding with water [26]. Moisture ingress in composites may impair mechanical properties due to the degradation of the fibers, consequently restricting their use to indoor applications only. Desirable, advanced outdoor applications of natural-fiber-reinforced composite and nanocomposite materials, however, can be achieved by reducing their water uptake and improving moisture resistance.

The aforementioned inherent limitations of natural fiber composites can be addressed either by using coupling/compatibilizing agents and/or pretreating natural fibers. Several pretreatment methods, such as physical (e.g., plasma or corona treatment and steam explosion) and chemical (e.g., NaOH/H_2_O_2_ treatment and acetylation) methods, have been reported [27,28]. Considerable reduction in water uptake of jute/polyester composites from the incorporation of glass fiber has also been reported [29]. A study revealed that incorporation of impermeable fillers into natural fiber composites is another effective approach to lower the moisture uptake of composites. It is well documented that filler shape, size, concentration, and distribution affect the barrier, mechanical, and thermal properties of the resulting composites [30,31]. In recent decades, there has been growing interest in the application of new platy-structured, nanofiller-filled materials represented by nanocomposites due to their high aspect ratio [32,33,34]. Among widely used platy-structured nanofillers, montmorillonite (MMT) is the most well regarded [35]. The simultaneous presence of two or more reinforcing/filling phases in a single matrix allows for the development of hybrid composites [17]. As each filler adds its characteristics to the host matrix, the incorporation of two fillers in a single matrix can compensate for the drawbacks of a single filler. There have been studies on the fabrication of hybrid fillers in a single matrix, and the enhanced properties of composites prepared by adding hybrid fillers have already been reported [36,37]. A literature review indicated that relatively few studies have been conducted to investigate the reinforcing effect of MMT in RH/polypropylene (PP) composites. In addition, the durability of composites against moisture exposure has seldom been reported.

The current study aimed to reveal the potential of MMT as a water-resistant reinforcing filler in PP/RH composites. Mechanical (tensile and flexural), morphological (SEM and XRD), and thermal (TGA and DSC) analyses were performed to examine the effectiveness of MMT incorporated in PP/RH composites. The effect of water uptake on the mechanical properties of the composites was also investigated.

## 2. Materials and Methods

### 2.1. Materials

MMT (1.30P), purchased from Nanomer^®^ (Nanocor Inc. Hoffman, IL, USA, was used as a water-resistant reinforcing filler. The montmorillonite (70 wt %) was modified with octadecylamine (30 wt %). RH, obtained from a local rice mill, was washed, dried overnight, and then ground. An air-circulating oven was used to dry the RH. Polypropylene was purchased from the local market. Polypropylene-grafted maleic anhydride (PP-g-MAH) was used as a commercial-grade compatibilizing agent. It was obtained from OREVAC^®^ (OREVAC^®^ CA100, Arkema, Colombes, France and had a melt flow index of 10 g/10 min and a density of 0.905 g/cm^3^.

### 2.2. Composite Fabrication

In this study, MMT was used as a water-resistant reinforcing filler and PP-g-MAH was used as a compatibilizing agent for a PP-based immiscible composite system. Composites with different contents were prepared and the formulations are summarized in Table 1.

**Figure 1:**
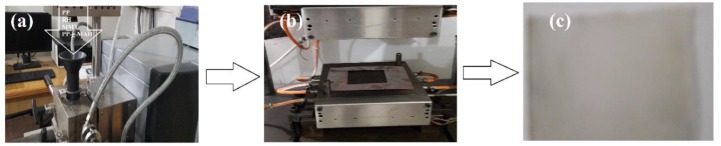
A schematic of the composite sheet fabrication process: (**a**) melt compounding in an internal mixer, (**b**) compression molding, and (**c**) compression-molded film sample for testing.

Weighed quantities of PP, RH, PP-g-MAH, and MMT were fed into an internal mixer (Haake Polylab OS, Thermo Scientific™, Waltham, MA, USA) for melt compounding. Melt compounding was carried out for 7 min at a 50 rpm rotor speed and 230 °C. The compounded composite lumps were compression-molded to prepare 0.3 mm thick sheets by pressing the samples at 200 MPa and 230 °C for 3 min. The compression-molded sheets were used to prepare samples for characterization. A schematic of the composite sheet fabrication process is shown in Figure 1.

### 2.3. Characterizations

#### 2.3.1. X-ray Diffraction (XRD)

In this study, XRD was used to calculate the delamination of MMT platelets in comparison to that of neat MMT (*d*_0_). A Bruker D8 Advance diffractometer was used in reflection mode to obtain XRD patterns. X-ray analysis was performed with an incident X-ray wavelength (*λ*) of 0.154 nm in the range of 2° < 2*θ* < 10° by a step of 0.02°. The interlayer distance in MMT was determined from the peak position in the spectrum using Bragg’s law (Equation (1)), and the relative intercalation (RI) of the PP in MMT was calculated using Equation (2):

(1) $$d=\frac{\lambda }{2Sin\theta }$$

(2) $$RI=\frac{d-d_{0}}{d_{0}}\times 100$$

where *d*_0_ and *d* are the MMT interlayer distances in neat MMT and its nanocomposites, respectively, and 2*ϴ* is the diffraction angle.

#### 2.3.2. Field Emission Scanning Electron Microscopy (FESEM)

Degree of filler(s) dispersion and their interfacial interactions with the polymer matrix were studied using FESEM (Hitachi S-4800, Tokyo, Japan. The fracture samples (cross section) were mounted on a standard specimen stub using double-sided sticky tape. Prior to scanning, the mounted samples were gold-coated using a BIO-RAD SEM coating system to avoid electrostatic charging, as uncoated samples may result in poor resolution during investigation.

#### 2.3.3. Water Absorption Tests

Water absorption tests of the prepared composite samples were carried out by immersing the samples in distilled water at room temperature. Water uptake of the samples was noted using a digital weighing scale, initially after 2 h of immersion and then after every 24 h of immersion for 10 days. The gain in weight of the composite samples due to absorbed water after time t was calculated as in Equation (3):

(3) $$W\left(\%\right)=\frac{W\left(t\right)-W\left(0\right)}{W\left(0\right)}\times 100$$

where W(0) and W(t) are the weight of the original dry sample and the weight of the immersed sample after time t. The mean of three data points was calculated and reported.

#### 2.3.4. Mechanical Properties

Dumbbell-shaped specimens were used for the tensile and flexural tests using Lloyd’s universal testing machine. Testing was carried out according to ASTM D638 and D790 standards for tensile and flexural properties, respectively. The samples were also immersed in distilled water. The immersed samples were soaked for 10 days at room temperature to compare their mechanical properties with dry samples. Prior to mechanical testing, the water-soaked samples were wiped to remove excess surface water. The average of five replicates and their standard deviation were calculated and analyzed for all mechanical tests.

#### 2.3.5. Thermal Properties

Thermal stability was investigated through TGA (Perkin-Elmer model TGA7 thermal analyzer) and DSC (Perkin-Elmer DSC-7). The testing was carried out from room temperature to 800 °C for TGA and room temperature to 220 °C for DSC. Samples were analyzed under a nitrogen environment, and a heating rate of 10 °C/min was adopted for both TGA and DSC. When a polymer crystallizes, it releases heat, so the enthalpy can explain the change of crystallinity [38]. Sample crystallinity (*X_c_*) was calculated by comparing PP heat of fusion (165 J/g) with that of composite samples using the following relation:

$$X_{c}=\frac{\Delta H}{\Delta H_{m}\cdot W_{PP}}\times 100$$

where

Δ*H_m_* = Melting enthalpy of 100% crystalline polypropylene (209 J/g [39]);
*W_PP_* = Weight fraction of PP in the composite sample.

## 3. Results and Discussion

### 3.1. X-ray Diffraction

**Figure 2:**
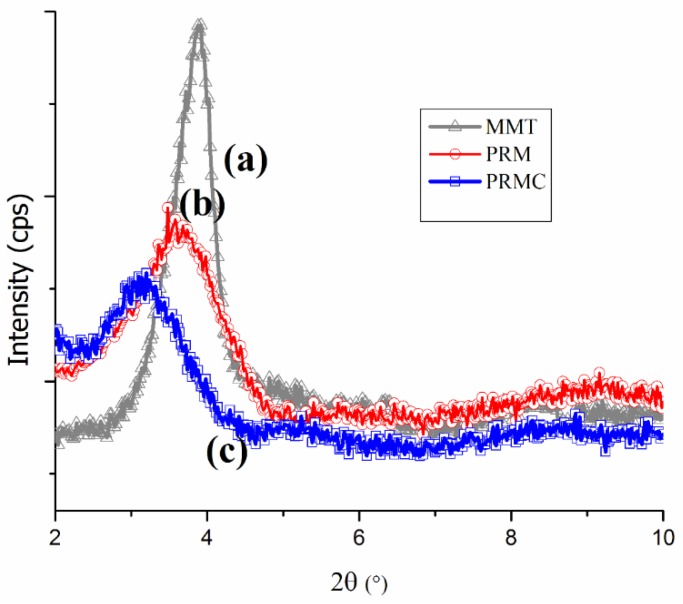
XRD diffractograms of (**a**) MMT, (**b**) PRM, and (**c**) PRMC.

Acceptable delamination of MMT in the host matrix due to the insertion of matrix chains in the interlayer of MMT governs the fabrication of MMT nanocomposites, as delaminated platelets have the potential to disperse in matrix. Figure 2 depicts the XRD diffractogram of the prepared nanocomposites.

As shown in Figure 2, the diffractograms suggest the formation of intercalated or semi-exfoliated composites due to the presence of a diffraction peak and their movement toward lower angles. Disappearance of the diffraction peak represents possible exfoliation of MMT platelets in the polymer. Changes in the interlayer spacing (d-spacing) of MMT owing to interlayer delamination, along with its relative intercalation, are summarized in Table 2.

The peak of the neat MMT was centered at about 3.90°, corresponding to the interlayer spacing of 2.26 nm. For the MMT-reinforced PP/RH nanocomposite system (PRM), the peak moved slightly toward lower 2θ at about 3.74°, indicating weak interactions between the MMT and PP that made it difficult for PP chains to enter into MMT galleries [13,40]. This slight change in 2θ may have been due to MMT platelet delamination by the shear stress in the extrusion process and, consequently, intercalation of PP chains into MMT galleries. The addition of a compatibilizer into the MMT-reinforced PP/RH nanocomposite system (PRMC) significantly increased the interlayer spacing, from 2.26 to 2.83 nm, with a 25% improvement in relative intercalation compared with neat MMT. The profound expansion of the MMT’s interlayer spacing was due to favorable interactions between the MMT’s interlayer moiety and the polymer chains, resulting in penetration and intercalation of PP chains into the MMT galleries. The increased delamination of MMT in the PRMC nanocomposite sample revealed the formation of intercalated structures [41]. Interestingly, all the MMT/PP/RH nanocomposites had higher interlayer spacing than neat MMT, and the noticeable improvement in the delamination of MMT platelets with the addition of a PP-g-MAH compatibilizer will lead to the development of nanocomposites with enhanced mechanical properties.

### 3.2. Field Emission Scanning Electron Microscopy

**Figure 3:**
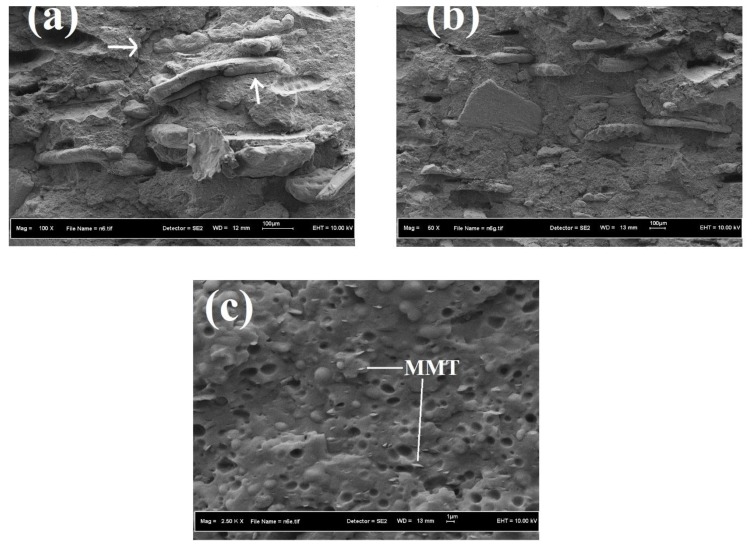
SEM micrographs of cryofractured surfaces of (**a**) PR, (**b**) PRC, and (**c**) PRMC.

FESEM is the most advanced and effective technique to investigate the dispersion, interfacial adhesion level, and morphology of solid particulates with polymer matrices. It played a vital role in describing the mechanical properties of the resulting composites, as displayed in Figure 3.

Figure 3a shows that the RH particles were not well dispersed in the PP and started agglomerating. The presence of hydroxyl groups could be a plausible reason for this agglomeration. In addition to these agglomerates, microvoids were also present between the RH and PP (shown by arrows in Figure 3a). The presence of RH aggregates and microvoids demonstrates the low compatibility and poor dispersion of the filler, thus necessitating physical/chemical pretreatment of RH and/or the addition of a compatibilizer into the composite system. Filler agglomeration not only decreased composite homogeneity but also resulted in void formation, which acted as stress concentrators. These stress concentrators led to early rupture of the composites due to the nonuniform stress translation. This poor interfacial bonding between the RH and PP and the presence of RH agglomerates can be attributed to the low polarity of PP and the high surface energy of RH [42]. However, the addition of a PP-g-MAH compatibilizing agent improved interfacial bonding between the filler(s) and the matrix material, as shown Figure 3b. Figure 3b clearly demonstrates that in the compatibilized composites, RH fibers were well dispersed and adhered to the PP matrix due to enhanced interfacial adhesion. The enhanced interfacial bonding favored the mechanical properties of the resulting nanocomposites. Enhanced dispersion of natural fibers and MMT with the addition of maleic-anhydride-grafted copolymers has also been reported by other researchers [43,44].

### 3.3. Water Absorption

**Figure 4:**
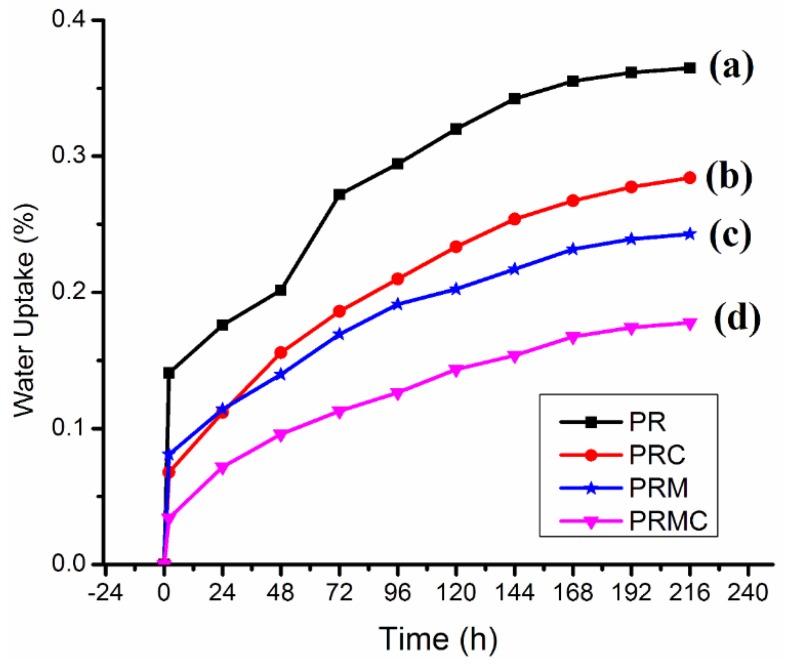
Water uptake of different composites: (**a**) PR, (**b**) PRC, (**c**) PRM, and (**d**) PRMC.

Natural fibers are prone to water uptake owing to the hydrophilic character of their major constituent, cellulose. This hydrophilicity can be associated with the presence of readily available hydroxyl groups. Due to the poor resistance of natural fibers toward water absorption, water absorption of natural-fiber-reinforced composites is among the key characteristics to be evaluated, as water absorption can lead to the degradation of mechanical properties. Figure 4 depicts and compares the percentage of water absorbed as a function of immersion time.

As expected, PR exhibited the highest water uptake in comparison with PRC, PRM, and PRMC. In addition, the water uptake of the different compositions had the following trend: PR > PRC > PRM > PRMC. It is worth noting that the rate of water uptake for all the prepared composites was dramatically high for first 2 h. After this time span, the degree of water uptake started gradually increasing with immersion time. PR exhibited the highest amount of water uptake, which can be associated with the hydrophilic character of RH. The presence of readily available hydroxyl groups in RH which can interact with water molecules might be responsible for this. The decrease in water uptake with the addition of PP-g-MAH can be attributed to the decrease in the amount of available hydroxyl groups capable of forming interactions with water molecules [45]. The MAH present in PP-g-MAH interacted with the hydroxyl groups of RH and PP chains; on the other hand, it interacted with the matrix. Consequently, there was a reduction in available hydroxyl groups capable of forming interactions with water molecules. The decrease in water uptake with the incorporation of PP-g-MAH suggests improved compatibility between RH and PP. Compositions with MMT absorbed less water, most probably due to the addition of water-resistant MMT. The decrease in water uptake may also have been due to the occupation of voids at the interphase between RH and PP and the obstruction of capillaries in RH by MMT [46].

### 3.4. Mechanical Properties

**Figure 5:**
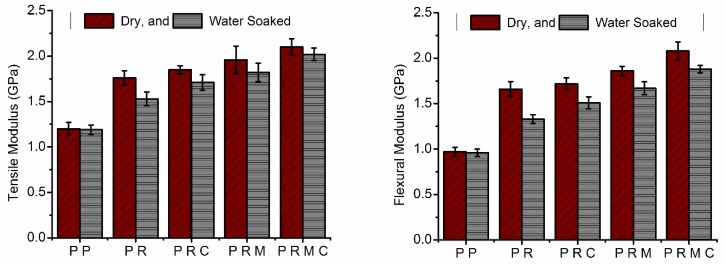
Tensile and flexural modulus of neat PP and its composites under dry and wet conditions.

**Figure 6:**
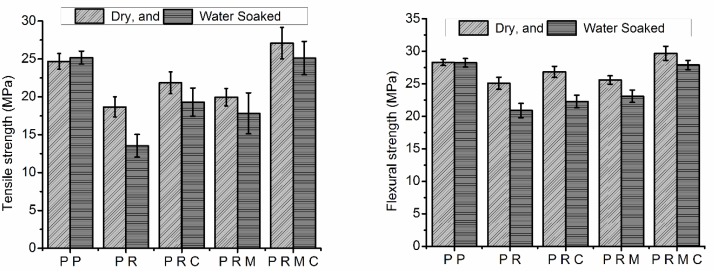
Tensile and flexural strength of neat PP and its composites under dry and wet conditions.

Comparative studies were made to explore the reinforcement effects of RH and MMT loadings and PP-g-MAH compatibilization on the tensile and flexural properties of the PP composites with the neat polymer. The effects of adding the reinforcing filler and the compatibilizer on the mechanical properties of both dry and water-soaked samples to validate the durability of these samples in humid/outdoor applications are displayed in Figure 5 and Figure 6.

Figure 5 depicts and compares the tensile and flexural modulus of compatibilized/uncompatibilized composites under dry and wet conditions. The tensile and flexural modulus of neat PP was 1.2 and 0.97 GPa, respectively, and a substantial improvement in the stiffness was observed with the incorporation of reinforcing filler(s) under both dry and wet conditions. This improvement was indicated by the tensile and flexural modulus of PP increasing by 47% and 71%, respectively, with the addition of RH (PR composites) and increasing by 63% and 92%, respectively, with the addition of RH/MMT simultaneously into the PP matrix. In the case of RH and MMT incorporated into PP, more deformation stress was required, as both RH and MMT are stiff and high-modulus materials. Therefore, the increase in PP tensile and flexural modulus with the addition of filler(s) can be ascribed to the rigidity of the fillers. Improvement in the PP modulus with the addition of natural fibers and/or MMT is also consistent with previous studies [34,47,48,49].

Figure 5 also reveals that adding a compatibilizer (PP-g-MAH) to uncompatibilized composite systems (PR and PRM) resulted in a subsequent increase in both the tensile and flexural modulus of the PRC and PRMC systems. This behavior was due to the improvement of the interfacial adhesion between RH or MMT and the PP matrix material. In addition, greater modulus values were achieved when MMT and PP-g-MAH were used in the fabrication of PP/RH-based nanocomposites. The tensile and flexural modulus of PP/RH composites increased by 5% and 4%, respectively, with the addition of PP-g-MAH and 36% and 25%, respectively, when using MMT in the presence of the compatibilizer. The increase in the modulus with the addition of MMT to the compatibilized composite system can be explained by the presence of delaminated, stiffer platelets and the high aspect ratio of MMT in the PP polymer matrix, which results in greater interaction within PP chains. The increase in modulus with the addition of rigid fillers in a polymer matrix has also been reported in the literature [13,34,40].

The absorption of water reduced the tensile and flexural modulus of all the composites except neat PP, which hardly absorbed any water, and this detrimental effect was quite observable. However, the magnitude of difference in the modulus values of both dry and water-aged samples decreased with the incorporation of the PP-g-MAH compatibilizer and further decreased with the addition of MMT to the composite system. The tensile and flexural modulus of PR decreased by 13% and 19%, respectively, and that of PRMC decreased by 7% and 10%, respectively, after soaking in water. The modulus damage of the composites from their water soaking could be due to the decreased stiffness of composite constituents. The damage may also have occurred due to differential swelling at the interphase. Differential swelling may lead to debonding between the fiber and matrix. This finding is also in agreement with other research reports [50].

Figure 6 shows the effect of filler(s) loading on the tensile and flexural strengths of uncompatibilized and PP-g-MAH-compatibilized PP composites under dry and wet conditions. Both the tensile and flexural strengths of the composites decreased with the RH loadings under both dry and wet conditions. The decrease in strength could have been due to the poor compatibility of RH with PP. Poor compatibility results in poor interfacial adhesion and, consequently, discontinuity in the matrix material. These findings are also in agreement with those of [42]. The addition of PP-g-MAH minimized the decrease in tensile and flexural strength by enhancing the compatibility of RH with the PP matrix by mediating the polarity between RH and the PP matrix in comparison with the uncompatibilized composites. The compatibilized composite samples (PRC) showed an increase of about 17% and 7% in tensile and flexural strength, respectively, compared with uncompatibilized composites (PR). PP-g-MAH enhanced the interfacial adhesion between polar RH and nonpolar PP through the interaction between the hydroxyl groups of RH and the carboxyl groups of PP-g-MAH. These results suggest better stress transfer from the matrix to the RH fibers, indicating improved interfacial bonding with a consequent improvement in the mechanical properties. In addition to this chemical interaction, PP chains of PP-g-MAH also diffused into the PP matrix, leading to the physical entanglement of PP molecules [48]. The maximum tensile and flexural strength values were found to be 27.1 and 29.7 MPa, respectively, for PRMC, while the tensile and flexural strength was approximately 19.9 and 25.6 MPa, respectively, for uncompatibilized PRM composites. These observations suggest that incorporation of MMT into the PP/RH hybrid system increased the tensile and flexural strength, and the extent of the improvement in the strength values was more pronounced in the presence of the PP-g-MAH compatibilizer due to the delamination of MMT platelets and their uniform dispersion in the polymer matrix. The improvement of the tensile and flexural strength with the addition of MMT into the PP/RH composite system is consistent with the SEM micrographs and XRD results.

The effect of prolonged moisture exposure on the tensile and flexural strength was quite considerable, and all the water-soaked composite samples showed lower tensile and flexural strength values compared with the dry composites. However, the extent of the decrease in strength values greatly depended on the composite constituents. The highest percentage decrease in tensile and flexural strength of water-soaked composite samples was observed for PR composites of about 27% and 17%, respectively, while the lowest decrease of about 7% and 6%, respectively, was observed for PRMC nanocomposites, relative to dry samples. The considerable decrease in the magnitude of the tensile and flexural strength of dry and water-soaked composites was primarily due to enhanced interfacial adhesion from the compatibilization effect of adding a compatibilizer between RH/MMT and the PP matrix along with the properties of MMT itself.

### 3.5. Thermal Analysis

**Figure 7:**
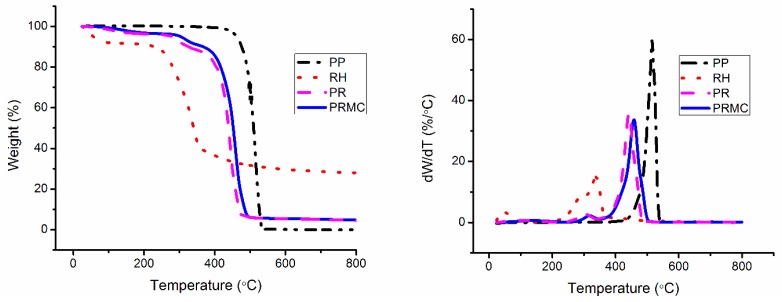
Thermogravimetry/derivative thermogravimetry (TG/DTG) curves for neat PP, RH, and the representative composites.

The thermal behaviors of neat PP, PP composites, and PP nanocomposites with or without a compatabilizer were analyzed by TGA. The thermal degradation temperature at 10% and 50% weight loss (T_10_ and T_50_) obtained by thermogravimetry (TG) and derivative thermogravimetry (DTG) scans are tabulated in Table 3. The TG and DTG curves for PP, RH, PR, and PRMC are shown in Figure 7.

As shown in Figure 7, the weight loss of PP followed a one-step degradation process, ranging from 425 to 550 °C. In contrast, the thermal degradation of RH followed a three-step (evaporation of water, degradation of cellulosic substances, and degradation of noncellulosic materials) degradation process, as indicated by TGA/DTG curves. The observed thermal behavior of RH was similar to that realized by other lignocellulosic fibers [51,52,53]. The initial degradation temperature for PP was 299 °C. Incorporation of RH into the PP matrix resulted in an increase in the degradation temperature of PP, which can be attributed to the lower thermal degradation of RH compared with that of PP. The degradation temperatures of RH-reinforced PP (PR) at 10% and 50% weight loss were 311 and 437 °C, respectively, and further addition of PP-g-MAH into the PP/RH (PRC) composites resulted in a small change in the decomposition temperature of PP/RH. This may be due to the better interaction between RH and the PP bridged by PP-g-MAH. However, improvement in the thermal stability of the PP/RH system was more prominent with the addition of MMT/PP-g-MAH. The marked improvement in the thermal degradation resistance of the PP/RH system with the addition of MMT/PP-g-MAH (PRMC) nanocomposites was due to the presence of delaminated and uniformly dispersed MMT platelets. In other words, MMT delamination and exfoliation in nanocomposites is crucial and account for the improvement in thermal degradation resistance [54]. It has been reported that delaminated, exfoliated, and better-dispersed MMT platelets account for the improvement in thermal degradation resistance [55]. Improved thermal stability with the addition of MMT may also be associated with the limited motion of polymer chains owing to dispersed MMT platelets. Rigid, impermeable MMT platelets are thought to reduce heat conduction. Thus, their presence limits the mobility of polymer chains [56].

Melting and crystallization peaks for neat PP were observed at about 163 and 117 °C, respectively, as shown in Table 3. The incorporation of RH into PP (PR) resulted in an increase in the crystallization temperature (T_c_) of the PP matrix. In addition, the incorporation also increased the *X*_c_ while maintaining the melting temperature (T_m_). This can be explained due to the nucleating ability of RH fibers for the crystallization of PP. An increase in the *X*_c_ of PP with the incorporation of cocoa pod husk fibers has also been reported by Chun et al. [39]. Moreover, the T_m_ remained unaltered with the further incorporation of PP-g-MAH and/or MMT. However, the T_c_ slightly decreased with the incorporation of MMT and/or PP-g-MAH. Concerning crystallinity, a slight increase was observed by the addition of MMT to the PP/RH composite system and the increase was more prominent with the addition of a compatibilizer, as in the case of the PRMC nanocomposites. The observed changes in the properties and the nucleation effect owing to the incorporation of MMT and PP-g-MAH to PP are inconsistent with other findings [57]. The increase in the crystallinity from the incorporation of MMT/PP-g-MAH probably had two causes: (1) the higher crystallinity of PP-g-MAH and/or (2) the enhanced interfacial adhesion between PP and MMT [40,44]. Thus, the enhanced crystallinity can be attributed to the intercalation of PP chains between MMT platelets and the possible interaction between them, where MMT platelets may act as nucleation sites. It is thought that impermeable crystalline regions enhance stress transfer, which influences the overall composite mechanical properties. Thus, the improvement in crystallinity enhanced the mechanical properties of the composite system under both dry and wet conditions.

## 4. Conclusions

RH- and MMT/PP-based composites and nanocomposites were successfully fabricated using twin-screw extrusion followed by compression-molding, and the effects of RH, MMT, and PP-g-MAH compatibilizer on the mechanical, morphological, and thermal properties were analyzed. SEM micrographs revealed that the compatibilizer helped PP chains penetrate and delaminate the MMT platelets to create a large filler aspect ratio. In addition, the compatibilizer enhanced natural fiber dispersion in the PP matrix and minimized RH aggregate and microvoid formation in the PRC and PRMC composite systems. The tensile and flexural modulus considerably improved; however, tensile and flexural strength decreased with the addition of RH to the PP matrix. Tensile and flexural properties were improved with the addition of MMT to the PP/RH system, and this phenomenon was stronger in the presence of the PP-g-MAH compatibilizer. The tensile and flexural modulus of PP/RH composites increased by 5% and 4%, respectively, with the addition of PP-g-MAH and 36% and 25%, respectively, when using MMT in the presence of the compatibilizer. Moisture absorption minimized the tensile and flexural properties; the highest decrease was observed for PP/RH composites (PR) and the lowest decrease was observed for MMT-reinforced and PP-g-MAH-compatibilized (PRMC) composites. Tensile and flexural strength decreased by about 27% and 17%, respectively, for PP/RH composites and by about 7% and 6%, respectively, for PRMC composites after 10 days of water soaking. The thermal stability of the composites decreased with the addition of RH, while the addition of MMT to PP/RH composites improved the thermal stability with respect to PP/RH composites. Moreover, DSC results revealed that the incorporation of RH, MMT, and PP-g-MAH to the PP matrix enhanced the crystallinity while maintaining the melting and crystallization temperatures.

## Figures and Tables

**Table 1:** Formulations and designations of the prepared composites.

Sample Designation	PP (wt %)	RH (wt %)	PP-g-MAH (phc) *	MMT (phc) *
PP	100	0	0	0
PR	80	20	0	0
PRC	80	20	5	0
PRM	80	20	0	4
PRMC	80	20	5	4

* Parts per hundred parts of composite. Abbreviations: PP—polypropylene, RH—rice husk, PP-g-MAH—polypropylene-grafted maleic anhydride, MMT—montmorillonite.

**Table 2:** Interlayer spacing of MMT and its relative intercalation in nanocomposites.

Sample Designation	2θ (°)	Interlayer Spacing (nm)	Relative Intercalation (%)
MMT	3.90	2.26	-
PRM	3.74	2.36	4
PRMC	3.12	2.83	25

**Table 3:** Thermal properties of neat PP and its composites.

Sample Designation	T_m_ (°C)	T_c_ (°C)	*X*_c_ (%)	T_10_ (°C)	T_50_ (°C)
PP	163.2	117.3	27.7	474	510
PR	162.9	120.1	29.6	311	437
PRC	163.1	119.2	30.1	318	441
PRM	163.0	116.1	29.9	315	440
PRMC	162.8	116.5	31.5	327	451
